# "Resurrection of clinical efficacy" after resistance to endocrine therapy in metastatic breast cancer

**DOI:** 10.1186/1477-7819-4-40

**Published:** 2006-07-05

**Authors:** Amit Agrawal, John FR Robertson, KL Cheung

**Affiliations:** 1 Professorial Unit of Surgery, City Hospital, University of Nottingham, Nottingham, NG5 1PB, UK

## Abstract

**Background:**

In a significant proportion of metastatic breast cancer (MBC) patients whose tumour has progressed within 6 months of endocrine therapy (*de novo *resistance), it is generally believed that the chance of achieving clinical benefit (CB) with further endocrine therapy is minimal.

**Methods:**

Data was retrieved from a prospectively updated database of metastatic breast cancer. Relevant data was exported to SPSS™ software for statistical analysis.

**Results:**

In oestrogen receptor (ER) positive MBC patients with assessable disease, CB was achieved in 159 (71.3%) (1^st ^line) patients. When these patients were put on further endocrine therapy, the CB rates were 63.2% (on 2^nd ^line), 46.1% (on 3^rd ^line) and 20% (on 4^th ^line) with a median duration of response (DOR) in those with CB of 22, 12, 11 and 15 months respectively. The remaining 64(28.7%) patients had *de novo *resistance on 1^st ^line endocrine therapy. Seventeen of these patients were treated with further endocrine therapy. The CB rates were 29.4% (on 2^nd ^line) and 22.2% (on 3^rd ^line) with a median DOR in those with CB of 22.7 months and 14 months respectively.

**Conclusion:**

The chance of further endocrine response continues to decrease with each line of therapy, yet CB is still seen with reasonable duration even with a 4^th ^line agent. In addition, further endocrine response, with long duration, can be seen in a significant proportion of patients who have developed *de novo *resistance to 1^st ^line endocrine therapy. The use of further endocrine therapy should not be excluded under these circumstances.

## Background

Recurrences after initial breast cancer treatment often manifest systemically as distant metastases. There is, thus, perpetual need to have effective systemic agents including endocrine agents to deal with them. Since the advent of tamoxifen in early seventies, several endocrine agents have been in vogue. These agents used in appropriate scenarios have better side-effect profile over cytotoxic agents. However, limitation to exploitation of this profile of the endocrine agents occurs due to the development of resistance after some duration of clinical efficacy.

To counter the effect of resistance to 1st line endocrine therapy, sequential use of endocrine therapy with chemotherapy were starting to be practised in early eighties [1]. Later, sequencing of endocrine agents was possible due to the apparent lack of cross-resistance between individual endocrine agents. Therefore, changing to a different type of endocrine agent when resistance develops to one can circumvent this problem to some extent and delay the need for cytotoxic chemotherapy.

The effectiveness of one or the other agents as 1^st ^and 2^nd ^line therapy for patients with metastatic breast cancer (MBC) has often been reported in literature. But there is dearth of reports of response to further lines of therapy. However, we have seen in our experience[2] that response to further lines of therapy is obtained even with development of resistance to 1^st ^and 2^nd ^line therapy. This **"resurrection of clinical efficacy" **appears to be largely an understatement in the present literature. We, therefore, report relative efficacy of further endocrine agents in our clinical series and aim to present an overview of available clinico-pathological characteristics of tumours in patients in this series who have had further clinical response following development of *de novo *and acquired resistance.

## Patients and methods

### Patients

Patients with MBC diagnosed and treated in our unit between 1994 and 2004 had clinical and pathological data collected prospectively and updated regularly on a database. In this study, patients who fulfilled the following criteria were investigated:

• Oestrogen receptor (ER) positive disease.

• With disease assessable by UICC criteria [3].

• First line therapeutic drug for metastatic disease was an endocrine agent in postmenopausal women with additional goserelin in premenopausal women.

• In the presence of ER positive disease, endocrine therapy was indicated at the outset due to the presence of non-visceral (predominantly bone only), non-rapidly progressive disease and as later lines of therapy in patients who have had clinical benefit with previous endocrine agents or were not fit or unwilling to receive chemotherapy in the presence of potentially life-threatening visceral metastases. Where the patients were fit and willing for cytotoxic chemotherapy in the presence of potentially life-threatening visceral metastases, further endocrine therapy was not given.

• On treatment for at least 6 months unless they progressed prior (in which case they were deemed to have *de novo *resistance).

• Patients were deemed to have progressed on endocrine therapy if the assessable lesions progressed or the patients developed new non-visceral or visceral lesions.

• When analysing the efficacy of endocrine agents in each line of treatment, data of patients not treated with endocrine agents in that particular line were excluded and also of those patients who were withdrawn earlier than 6 months owing to side-effects or those with incomplete treatment data or follow-up data.

### Use of bisphosphonates

• Bisphosphonates were used in women with bony metastasis as per local guidelines. The agents used were Pamidronate/Zoledronate and Clodronate.

### Assessment of therapeutic response

• This was made as per UICC criteria. Assessable lesions were deemed to have shown clinical benefit (CB) when they either had objective response in the form of complete response (CR) or partial response (PR); or had stable disease (SD) for ≥ 6 months [4,5].

• Duration of response (DOR) is the duration of treatment of patients who have derived CB.

• Disease-free interval (DFI) is defined as the duration from the date of treatment for the primary cancer to the date of the first relapse regardless of its site.

### Definitions of endocrine resistance

• *de novo resistance *– Patients whose disease progressed within 6 months of treatment (i.e., never had CB from the endocrine agent).

• *Acquired resistance *– Patients who progressed after at least 6 months on treatment (i.e., having had CB from the endocrine agent).

### Methods

Two hundred and twenty three patients were eligible for study. Available clinical and pathological data of these patients was exported from the database onto SPSS™ software (SPSS Inc. USA).

The degree of linear relationship between duration of response and various variables was ascertained by Pearson's product-moment correlation coefficient (r). Bivariate correlation was considered to be significant at 0.01 levels (2-tailed). Correlation was direct with positive coefficient and inverse with negative coefficient.

## Results

The comparison of clinico-pathological characteristics between patients with *de novo *and acquired resistance to 1^st ^line endocrine therapy is shown in Table 1. The differential use of endocrine agents in first 5 lines of therapy is shown in Table 2A. The relative use of LHRH agonist (Goserelin) when given to premenopausal women in addition to other endocrine agents is shown in Table 2B.

**Table 1:** Comparison of clinico-pathological characteristics between patients with *de novo *and acquired resistance to 1^st ^line endocrine therapy

	***Acquired resistance***		***de novo resistance***	
Total = 223 (100%)	To 1^st ^line		To 1^st ^line	
CB (Clinical Benefit)	159 (71.3%)		64 (28.7)	
Median duration of treatment in months	15.7+		2.9	
Median Age (Range) at metastasis in years	63.5 (30–89)		61.5 (32–87)	
DFI (months)	58.0 (0–294)		36.5 (0–324)	
Median Age (Range) at primary cancer in years	56.0 (26–86)		55.0 (28–87)	
Maximum Size (Range) of tumour in mm.	24 (4–110)		27 (10–110)	
Minimum Size (Range) of tumour in mm.	20 (2–70)		25 (10–75)	
Histological lymph node stage(no. of positive nodes)	1 (0)	39.8	1 (0)	37.5
	2 (1–3)	41.8	2 (1–3)	37.5
	3 (≥4)	18.4	3 (≥4)	25.0
Grade (Elston/Ellis)	1	11.6	1	9.3
	2	49.3	2	37.0
	3	30.4	3	40.7
	unknown	8.7	unknown	13.0
Vascular invasion	44.1%		50.0	
Bony metastasis	61.5		60.3	
Lung metastasis	21.4		23.9	
Pleural metastasis	25.5		47.8	
Lymphangitis	2.1		15.2	
Liver metastasis	10.4		41.8	
Other metastasis	35.2		28.8	

+ = including patients who are still on treatment, therefore would have additional duration of response on a later analysis; DFI = Disease free interval

**Table 2A:** Relative use of various endocrine agents

**N (%)**	**1^st ^line 223 (100)**	**2^nd ^line 115 (100)**	**3^rd ^line 71 (100)**	**4^th ^line 32 (100)**	**5^th ^line 8 (100)**
Tamoxifen	85(38.1)	20(17.4)	5(7.0)	4(21.5)	2(25)
Anastrozole	110(49.3)	59(51.3)	12(17.0)	2(6.2)	2(25)
Exemestane	6(2.7)	24(20.9)	18(25.3)	18(56.3)	3(37.5)
Megestrol	5(2.7)	10(8.7)	35(49.3)	6(18.8)	None
Fulvestrant	17(7.6)	2(1.7)	1(1.4)	2(6.2)	None
Letrozole	None	None	None	None	1(12.5)

**Table 2B:** Relative use of LHRH agonist (Goserelin) in premenopausal patients in addition to other endocrine agents

**Patients with Goserelin/Total patients treated each line (%)**	**1^st ^line 31/223 (13.9)**	**2^nd ^line 5/115 (4.3)**	**3^rd ^line 2/71 (2.8)**	**4^th ^line 2/32 (6.2)**	**5^th ^line 0/8 (0)**
Tamoxifen	12 (5.4)	None	None	1 (3.1)	None
Anastrozole	19 (8.5)	3 (2.6)	None	None	None
Exemestane	None	2 (1.7)	2 (2.8)	1 (3.1)	None

Out of a total of 223 patients, the majority (71.3%) of patients have had CB from 1^st ^line endocrine therapy as shown in Table 3A. The majority of patients who developed acquired resistance following CB to 1^st ^line endocrine therapy went onto subsequent endocrine therapies with further demonstrable CB as shown in Table 3B.

**Table 3A:** Clinical benefit achieved from 1^st ^line endocrine therapy in all patients

***Endocrine therapy***	***1^st ^line***
N	223
**N of CB (%)**	**159 (71.3%)**
Median DOR with CB (months)	22+(7–117)
N still receiving treatment	50

+ = including patients who are still on treatment, therefore would have additional duration of response on a later analysis; CB = Clinical Benefit; DOR = Duration of Response

**Table 3B:** Clinical benefit achieved from subsequent lines of endocrine therapy in patients with clinical benefit to 1^st ^line endocrine therapy

***Lines of Endocrine therapy***	***2^nd ^line***	***3^rd ^line***	***4^th ^line***
N	68	23	5
**N of CB (%)**	**47 (69.1)**	**10 (43.5)**	**1(20.0)**
Median DOR with CB (months)	12.0+(7–81)	11.0 (8–32)	15.0
N still receiving treatment	17	0	0

+ = including patients who are still on treatment, therefore would have additional duration of response on a later analysis; CB = Clinical Benefit; DOR = Duration of Response

In those patients who had *de novo *resistance to 1^st ^line endocrine therapy but still went on to 2^nd ^line endocrine therapy, almost a third (29.4%) demonstrated CB with a median DOR being 12.0 (7–29) months. The CB to subsequent therapies following *de novo *resistance to 1^st ^line therapy is demonstrated in Table 4A. Further, among the 21 patients who had *de novo *resistance to 2^nd ^line following CB to 1^st ^line endocrine therapy, 6 patients were put on further lines of endocrine therapy with responses as demonstrated in Table 4B.

**Table 4A:** Clinical benefit achieved from subsequent lines of endocrine therapy in patients with *de novo *resistance to 1^st ^line endocrine therapy

***Lines of Endocrine therapy***	***2^nd ^line***	***3^rd ^line***	***4^th ^line***
N	17	9	4
**N of CB (%)**	**5 (29.4)**	**2 (22.2)**	**0**
Median DOR with CB (months)	12.0 (7–29)	14.0+(11–17)	Not applicable
N still receiving treatment	0	1	0

+ = including patients who are still on treatment, therefore would have additional duration of response on a later analysis; CB = Clinical Benefit; DOR = Duration of Response

**Table 4B:** Clinical benefit achieved from subsequent lines of endocrine therapy in patients with *de novo *resistance to 2^nd ^line endocrine therapy following clinical benefit to 1^st ^line endocrine therapy

***Lines of Endocrine therapy***	***3^rd ^line***	***4^th ^line***
N	6	2
**N with CB (%)**	**3 (50.0)**	**1 (50.0)**
Median DOR with CB (months)	15.0 (13–32)	9.0
N still receiving treatment	0	0

CB = Clinical Benefit; DOR = Duration of Response

Further analysis involved comparison between certain available pathological characteristics of the *de novo *resistant and acquired resistant groups (as in Table 1) in terms of the DOR. The median DOR of patients on 1^st ^line endocrine therapy correlated positively (r = 0.01) with the length of DFI but correlated inversely (r = 0.01) with grade 3 tumours; pre-dominant presence of pleural metastasis, lymphangitis and liver metastasis.

## Discussion

As shown in previous randomised studies, majority of the patients in our study had CB to 1^st ^and 2^nd ^lines of endocrine therapy. In addition, we demonstrate further response to 3^rd ^and 4^th ^line endocrine therapy after CB to previous 2 lines. The median DOR in all the 4 lines was shown to be almost a year with the 1^st ^line reaching 2 years.

Similarly, a proportion of patients who were treated with 2^nd ^line endocrine therapy in spite of *de novo *resistance to 1^st ^line endocrine therapy showed CB (almost a third) with a median DOR of a year. They were treated with further endocrine therapy either because of their individual choice or because they had non-visceral (predominantly bone only), non-rapidly progressive disease. A further proportion of patients who were treated with 3^rd ^line endocrine therapy after *de novo *resistance yet again to 2^nd ^line had CB with a median DOR of more than a year. However, there was no CB to 4^th ^line therapy after lack of CB to 1^st ^three lines of endocrine therapy.

The rate of CB decreased with subsequent endocrine therapy but responses were still seen to all 4 lines of therapy irrespective of either acquired or *de novo *resistance to previous lines (except to 4^th ^line with prior *de novo *resistance) though the number of patients was small by that time. Thus, "ultimate hormone resistance" does not appear to occur in this series despite several (up to 4 lines) of endocrine therapy. Furthermore, when there is response, sustained duration of CB is possible.

Although there was no statistical significance of the various tumour or metastatic characteristics in predicting response but they are suggestive of a trend especially in relation to the prior DFI of the MBC patients. As seen in the results, CB was obtained on 1^st ^line of endocrine therapy in those patients who had longer DFI and this was true even with patients who were put on 2^nd ^line endocrine therapy in spite of developing *de novo *resistance to 1^st ^line. This is suggestive of an inherent, yet undefined biological characteristic of the primary tumours which lends it longer DFI and subsequent enhanced responsiveness to endocrine agents at a later metastatic stage.

### Mechanism of development of resistance

Thus very similar to our results, it is known in literature that in-spite of significant proportion of MBCs being ER and/or progesterone receptor (PgR) positive, not all of them respond to endocrine therapy suggesting underlying intrinsic properties of the tumour and the underlying mechanisms of intrinsic resistance i.e., de novo resistance [6].

Various mechanisms of development of resistance have been suggested by different scientific groups. Dowsett [7] attributed the cause of *de novo *resistance to the presence of only very low levels of ER and presumed growth dependence on other pathways. However, with acquired resistance, he noted that the intra-tumoral concentration of tamoxifen was substantially reduced at relapse, despite no change in plasma levels. Hardin et al [8] demonstrated that dehydroepiandrosterone sulphate (DHEAS) exposure, even in the presence of tamoxifen and fulvestrant, induced changes in ER and PgR gene expression that may be partially responsible for breast cancer progression.

Thus, the mechanisms of development of resistance are from clear but there are ongoing efforts to develop markers or to identify tumour characteristics which may help predicting type of response to endocrine

### Predictive markers for type of response to endocrine therapy

In spite of their limitations, tumour grade and ER status have been reported as independent predictors of response to 1^st ^line [9,10] and 2^nd ^line [11] endocrine therapy. Recently Bardou et al [12] have shown that PgR status is an independent predictive factor for benefit from adjuvant endocrine therapy and improves outcome prediction of ER status.

Arpino et al [13] demonstrated in their study that patients with HER-2 amplification and HER-1 expression had lower ER levels and were modestly less responsive to tamoxifen, suggesting that molecular events in addition to those involving the ErbB receptors are important in determining the endocrine-resistant phenotype.

Significantly lower rate of response to 1^st ^line endocrine therapy was seen in patients with high vascular endothelial growth factor (VEGF) levels (P = 0.025) [14] and high levels of complex of urokinase-type plasminogen activator and its main inhibitor (P = 0.018) [15] when compared with lower levels.

Jansen et al [16] recently demonstrated differential expression of 81 genes (P < or = 0.05) between tamoxifen-responsive and resistant tumours in a study performed on 112 ER positive primary breast carcinomas from patients with advanced disease and clearly defined types of response (i.e., 52 patients with objective response versus 60 patients with progressive disease) from start of 1^st ^line treatment with tamoxifen. Gene expression profiling may thus be used to predict differential response to endocrine agents.

Accurate prediction of the type of response to endocrine therapy would simplify the future planning and management of MBC patients. Predictive markers can extend from simple and available clinical indicators such as ER, PgR or to some extent DFI as suggested in our dataset to more complicated but specific gene expression profiling. However, till highly sensitive and yet specific predictive markers are available, optimal sequencing of endocrine agents' based on available clinical data in literature has to be practised.

### Optimal sequence of endocrine agents

With the availability of different endocrine agents, the concept of sequencing becomes important so that we can ensure optimal use of agents while maintaining hormone sensitivity to subsequent agents which might act via different mechanisms. Newer agents which challenge the "gold standard" tamoxifen include 3^rd ^generation aromatase inhibitors and oestrogen downregulators such as fulvestrant. Further, endocrine response has been seen after fulvestrant has failed [17-19].

Traditionally, further endocrine therapy would only be considered when CB has been seen with a prior agent. This study demonstrates that further endocrine response could still be seen in a significant proportion of patients who have developed *de novo *resistance to 1^st ^line endocrine therapy. This is an important observation to be borne in mind when sequencing of endocrine agents is considered.

## Conclusion

The chance of further endocrine response continues to decrease with each line of therapy, yet CB is still seen with reasonable duration even with 3^rd ^and 4^th ^line agents; and even after *de novo *resistance is apparent with prior endocrine agents.

The present study, therefore, exposes sufficient clinical efficacy of further endocrine agents and "resurrection" of their response even after the tumours have had *de novo *resistance to previous lines of endocrine therapy. Based on our finding, we would like to re-iterate that further lines of endocrine agents should not be excluded in those oestrogen receptor positive MBC patients who have not had CB to previous endocrine agents. They may be particularly of benefit when these patients are not fit for aggressive chemotherapy or not keen on chemotherapy pending availability of simple yet accurate endocrine response predictive markers or the availability of utopian resistance-proof pharmaceutical product. This clinical study may be instigative in initiation of larger prospective studies aimed at defining intrinsic biological characteristics including genetic profiling. This pre-treatment definition of tumour may be predictive of the nature of response to planned endocrine therapy.

## Conflict of interest statement

We, the authors are fully conversant with and approve of the contents of the manuscript and have no potential or actual commercial, financial or political interest in the material.

## Authors' contributions

**JFR **and **KLC **conceived this study. Patients were under care of JFR and KLC. **AA **collected data, performed analysis, drafted, revised and finalised the manuscript. **KLC **and **JFR **revised and approved of the contents of the manuscript. All authors read and approved the final manuscript.
